# Creatively Adapting Touch-Based Practices to the Web Format During the COVID-19 Pandemic: Systematic Review

**DOI:** 10.2196/46355

**Published:** 2023-10-26

**Authors:** Greta Gauhe, Rosemary E Kostic Cisneros, Jade Ward, David J Hohenschurz-Schmidt

**Affiliations:** 1 Centre for Dance Research Coventry University Coventry United Kingdom; 2 Pain Group Department of Surgery & Cancer Imperial College London London United Kingdom; 3 Research Department University College of Osteopathy London United Kingdom

**Keywords:** digital health, telehealth, eHealth, touch, creative arts, web, digital therapeutics, manual therapy, psychotherapy, arts therapy, qualitative study, web delivery, implementation, barrier, physiotherapist, psychotherapist

## Abstract

**Background:**

The COVID-19 pandemic forced numerous touch-based fields, including manual therapy, body psychotherapy, arts therapy, creative arts practices, and the fitness industry, to swiftly transition to web-based service delivery. These disciplines faced substantial challenges in adapting their traditionally in-person practices, which rely heavily on physical touch and close proximity, to a web format.

**Objective:**

This review intends to provide a systematically sourced overview of the literature concerning innovative approaches for adapting touch-based practices to the web format in response to the COVID-19 pandemic.

**Methods:**

A systematic search across 7 databases and gray literature sources identified studies presenting innovative web delivery methods, particularly those addressing the challenges arising from the absence of physical proximity and touch. The inclusion criteria were designed to encompass studies exploring the creative adaptation of touch-based practices to web formats in response to the COVID-19 pandemic irrespective of study methodology. The exclusion criteria applied to studies focusing solely on technical aspects of web delivery or nontouch or noninteractive practices. There were no geographical restrictions, but the selection was limited to publications from 2020 onward. As only qualitative studies were found, data synthesis was conducted thematically.

**Results:**

The review encompassed 17 studies revealing that touch-based fields successfully devised innovative and creative methods for web service delivery. These methods were categorized into five main themes: (1) adapted working methods (cross-field methods), (2) adapted working methods for sensorial experiences and nonphysical connections, (3) creative methods using materials or additional tools, (4) creative use of web-based platform tools or additional technologies, and (5) creative methods requiring previous preparation of practitioners or participants. These creative approaches allowed practitioners to address the challenges of web touch-based practices, fostering connections and offering unique sensory experiences, albeit with some concerns related to technology and preparation.

**Conclusions:**

These innovative methods demonstrate promise in circumventing the limitations imposed by the lack of physical touch and proximity in web settings during the COVID-19 pandemic. Furthermore, these insights hold potential for application in other fields in the future. This systematic search and thematic synthesis provide valuable guidance for practitioners and educators seeking to navigate the evolving landscape of web service delivery in touch-based disciplines, ensuring continuity of care even in challenging circumstances.

**Trial Registration:**

PROSPERO CRD42022379731; https://www.crd.york.ac.uk/prospero/display_record.php?RecordID=379731

## Introduction

### Background

During the COVID-19 pandemic, many professionals had to adapt face-to-face working methods to suit web-based formats. This adaptation was particularly challenging in fields that normally rely on affective touch and physical closeness, such as manual therapy and other health care disciplines, art therapy, many creative arts practices, and the fitness industry. Nonetheless, these fields rapidly implemented different methods to ensure access to services and care. Instead of face-to-face meetings, manual therapists delivered telehealth consultations [1], fitness coaches and movement-based practitioners taught classes on the web [2], and midwives offered internet-based assessments [3]. Sometimes, practitioners built on existing experiences in their field (eg, in primary care [4]), and sometimes they explored web-based formats anew or learned from other fields [5]. Quickly, field-specific recommendations on remote care delivery and the use of ITs were published [5-9]. Although these publications acknowledged the challenge of the lack of physical closeness and touch-based practices in web-based settings, they rarely provided concrete resolutions to address this crucial aspect in remote sessions [8].

This review aimed to bring together diverse touch-based fields to examine and compare their distinct approaches for web-based practices. Despite their unique scopes of practice, aims, and session lengths, these fields share a common feature—the use of affective touch to foster trust-based relationships. Before the pandemic, touch played a vital role in interactions within these fields. For instance, manual therapists, nurses, and midwives used touch for physical examinations, hands-on interventions, and displaying empathy and compassion [10]. In dance therapy and practice, physical proximity is integral to interactions and serves as an artistic tool. Choreographers, physiotherapists, and fitness coaches used touch to convey instructions and provide physical support [11-13]. Amid the variations in practices, touch proved to be a powerful tool, enriching the overall experience for participants and patients alike.

Recent research in health shows that remote consultations are largely as safe as their face-to-face counterparts—and there is some evidence indicating comparable clinical effectiveness [14,15]. However, the satisfaction levels of both participants and practitioners varied. Artists, for instance, encountered technological and financial challenges when delivering performances on the web, whereas manual therapists were resistant to the concept of hands-off care and patients expressed unmet expectations [16,17]. Conversely, benefits such as improved accessibility and lower costs are frequently highlighted by both health practitioners and participants of web-based fitness classes [5,18,19].

Many touch-based professionals face similar challenges when delivering services on the web. Building a sense of allegiance expressing empathy becomes more complex in internet-based settings, as does effectively communicating on emotionally profound subjects [17]. Nevertheless, web-based and telephone interventions have become firmly established in various fields, even in emotionally sensitive areas such as psychotherapy for posttraumatic stress disorder [20]. The success of remote consultations is often influenced by preexisting relationships as familiarity with the clinician can enhance the effectiveness of the interaction [21]. Furthermore, manual therapy practitioners also grapple with the complexities of touch during in-person sessions as touch can trigger both positive and negative associations in patients [22]. Although web-based or remote sessions may offer increased accessibility and could be preferable for some individuals compared with direct in-person touch, the existing literature currently lacks information on the adverse effects of simulated touch in web-based sessions. This area requires further investigation in future studies.

There is preliminary evidence suggesting that individual practitioners and initiatives approach these challenges in innovative ways by making use of a variety of creative approaches. A web-based integrative oncology program, for example, implemented breathing exercises with a spiritual care provider, and the visualization of previous touch-involving treatments has been a success for remote consultations [6].

Finally, beyond the immediate public health emergency of COVID-19, there is an ongoing need to enhance currently used web-based methods [4]. The growing momentum of digital health interventions, as seen in the UK National Health Service plans for digital health and social care, exemplifies the significant potential for integrating technology in touch-based practices [20,23]. Telehealth is likely to become increasingly dominant in health care systems [15], especially in light of limited resources and for hard-to-reach populations or people with reduced mobility [21,22]. Arts performances and communication events such as conferences routinely happen on the web or as hybrid formats. Finally, screen-based fitness or movement classes are firmly established [24], and web-based learning and internet-based meetings have become the norm.

### Aims and Objectives

This review aimed to provide a systematically sourced overview of the literature concerning creative approaches for adapting touch-based practices to the web-based format in response to the COVID-19 pandemic.

## Methods

### Overview

A systematic review was performed as it offers a recognized methodology for the identification, appraisal, and synthesis of the best available evidence [25]. The review protocol was prospectively registered on PROSPERO (CRD42022379731).

The focus of this review was creatively adapted working methods from all touch-based fields to suit the new web-based format. For the review, adapted methods were considered “innovative” and “creative” if they took into account the specific challenge of the lack of proximity and lack of affective touch in web-based settings and aimed to achieve a similar outcome or goal as when meeting people in person. Furthermore, methods that were transferred without adaptation from in-person sessions to web-based meetings within the same profession were not deemed creative or innovative. We specifically focused on working methods implemented during the COVID-19 pandemic, assuming that the sudden and drastic disruption of in-person practice incubated most innovation from practitioners.

### Eligibility Criteria

To be included in this review, studies had to explore the web-based adaptation of an artistic, therapeutic, or otherwise interactive practice that usually relies on affective touch from providers or physical closeness with participants. “Affective touch” describes slowly moving, low-force touch that is often perceived as pleasant (as opposed to instrumental touch such as that used by surgeons) [24,25]. Regarding categorization, the review was expected to include physical therapists, manual therapists, fitness coaches, dance artists, dance therapists, body psychotherapists, and art therapists.

Furthermore, articles were included that presented innovative and creative adapted working methods to suit the web-based format, not just the attempt to transfer offline working methods to the web without specific adaptation of the otherwise hands-on element. For example, physiotherapists adapted their ways of working creatively if they used touch to identify an area of pain during face-to-face sessions yet, on the web, they would ask for assistance from a housemate to locate the area of pain instead [19]. The Oxford Dictionary defines “creative” as relating to or involving the use of the imagination or original ideas to create something. In the case of this review, adapted methods were deemed “creative” if they considered the specific challenges of lack of proximity and lack of affective touch in web-based settings and aimed to achieve a similar outcome or goal as when meeting people in person.

Given the variety of relevant fields and potentially relevant research designs, any study design was included.

The exclusion criteria for the review were studies that only reported on the technical aspects of web-based delivery and did not focus on the creative adaptations or the experiences of practitioners or participants. Studies that solely focused on the web-based delivery of nontouch or noninteractive practices were also excluded.

No geographic restrictions were applied, and primary outcome reports had to be published from 2020 onward. Since 2020, the active time frame during the pandemic has likely driven the increased development and implementation of creative methods for web-based settings. As identifying such methods from a vast and diverse literature can be challenging, we seized the opportunity presented by this brief but dynamic period to uncover and explore these innovative approaches. Publications in English and German were eligible, and those in other languages were also included if translations could be obtained. Studies were excluded if no full text could be retrieved either on the web or through the corresponding author. Textbox 1 summarizes all inclusion criteria.

**Eligibility criteria for the systematic review.**

**Inclusion criteria**

- Topic: articles must relate directly to the research questions and present creatively adapted working methods to suit the web format, not just the attempt to transfer offline working methods on the web without specific adaptation of the otherwise hands-on element (adapted methods were considered “creative” if they acknowledged the specific challenge of a lack of proximity and lack of affective touch in web settings and developed or implemented solutions with the aim of achieving a similar outcome as when meeting people in person)
- Population: all fields that usually rely on the application of affective touch from practitioners or physical closeness with participants
- Recency: published in or after 2020
- Geographical spread: no geographic restrictions
- Research base: all research designs
- Language: English or German; others if translations could be obtained
- Transparency: methodology of the research should be explicit (eg, sample sizes, instruments, and analysis)

**Exclusion criteria**

- Not complying with the aforementioned inclusion criteria

### Information Sources and Search Strategy

A systematic search was carried out in November 2022 in the following databases: MEDLINE, APAPsych, CINAHL, SPORTDiscus, the International Bibliography of Theatre and Dance, and AMED. These databases were selected to include the most relevant databases across a variety of fields that usually rely on physical closeness. Search terms were adapted to individual databases, and librarian support was obtained. The search strategy followed the Population, Intervention, Comparison, and Outcome (PICO) framework as presented in the Cochrane Handbook for Systematic Reviews of Interventions [23] and was developed under consultation with published literature, experts who are part of the research team (health, arts, and sociology researchers), and an expert in systematic review methodology. Indexing and free-text terms were used in combination. Following the suggestion by Butler et al [26], we used a modified version of the PICO framework to organize our research question and establish inclusion criteria. Multimedia Appendix 1 presents the search strategy for all databases.

The search terms covered the concepts “touch” AND “intervention terms” AND “profession names.” The complete search string can be found in Multimedia Appendix 1. Following iterative testing of the search strategy, terms such as “haptics,” “tactile,” and “palpation” yielded unresponsive search results. Consequently, only the term “touch” was retained and used in the final search strategy. Multimedia Appendix 1 shows how the PICO tool was used to structure the research question and search terms.

In addition, to capture all relevant work across this large variety of fields and as many artists do not publish their research in academic journals, gray literature was searched manually through Scopus, Google Scholar, the Google search engine, and Academia [27]. Searching for gray literature was similarly structured around the concepts of “adapted working methods during the pandemic,” “telemedicine AND touch,” “remote OR online AND dance performance,” and “artists AND adapted working methods AND pandemic.” Known arts organizations were also searched for specifically, and relevant links were followed up on (eg, from blog entries or news articles to artists’ primary websites). Furthermore, a librarian at the Jerwood Library of the Performing Arts in London helped identify relevant arts-related resources from their collection. Finally, the reference lists of the included articles were searched [28].

### Selection Process

Before screening, all references resulting from the database search were imported into EndNote (Clarivate Analytics), and duplicates were removed.

Title and abstract screening was conducted in duplicate and in a blinded manner to the parallel reviewer’s judgment. In total, 2 reviewers (GG and JW) screened the remaining articles using the Covidence platform (Veritas Health Innovation) for systematic reviews [29]. In a first step, titles and abstracts were screened against the inclusion and exclusion criteria in Textbox 1. Next, the remaining articles were accessed in full and screened again by 2 independent reviewers (GG and DJH-S). Conflicts were resolved through discussion and agreed upon through consensus.

After identifying studies via the other aforementioned methods, all additional records consisting of academic dissertations and published academic articles were recorded. These were screened by 2 reviewers and examined using the same inclusion criteria as for the database search screening. The remaining records were assessed for eligibility and included in the review. By appraising each study against the same criteria and recording the results, the basis for the review’s conclusions were made transparent.

### Data Collection

A data extraction form was created to capture pertinent information related to this review’s research questions. To ensure its effectiveness, a draft extraction form was tested on 3 papers by 1 researcher (GG) and refined accordingly. Data extraction was then carried out by 1 reviewer (GG) and cross-checked by another reviewer (DJH-S) using the standardized form, covering aims, study details, design, data collection, setting, study population, field of study, and country. The cross-checking process involved comparing the extracted data with the original sources to identify any discrepancies or variations, which were collaboratively resolved by the 2 reviewers. This quality assurance step strengthened the reliability and credibility of this review’s findings, ensuring accurate data synthesis and analysis.

The following information was extracted from the included articles: author or authors, year of publication, research aim, design and data collection methods, study population, setting, country, field of study, practitioners’ backgrounds, telehealth methods, original method that underwent adaptation, participants’ experiences with adapted working methods, practitioners’ experiences with adapted ways of working, requested improvements for web-based sessions, adapted working methods (cross-field methods and adapted working methods for sensorial experiences and nonphysical connections), and tips to increase telehealth acceptability.

### Quality Appraisal

The methodological quality of the included studies was appraised using the tools of the Critical Appraisal Skills Programme (CASP), allowing for separate assessment of qualitative, quantitative, and mixed methods studies.

The CASP tools do not have a numeric rating system as their primary aim is to promote a rigorous and comprehensive critical appraisal of research studies without oversimplifying the process. The CASP tool for appraising qualitative studies is divided into 10 sections: statement of aims, methodology, research design, recruitment strategy, data collection, research-participant relationship, ethical issues, data analysis, and findings valuable to us. Quality assessments were completed by 1 author (GG) and cross-checked by another author (DJH-S) to ensure accuracy.

### Synthesis

Thematic synthesis [30] was used, which is an approach that mirrors the thematic analysis used in primary qualitative studies. In brief, thematic synthesis is a 3-stage process that moves iteratively between the coding of text identified as relevant from primary studies, the identification of descriptive themes, and the generation of analytical themes across the analyzed studies [30].

**Figure 1:**
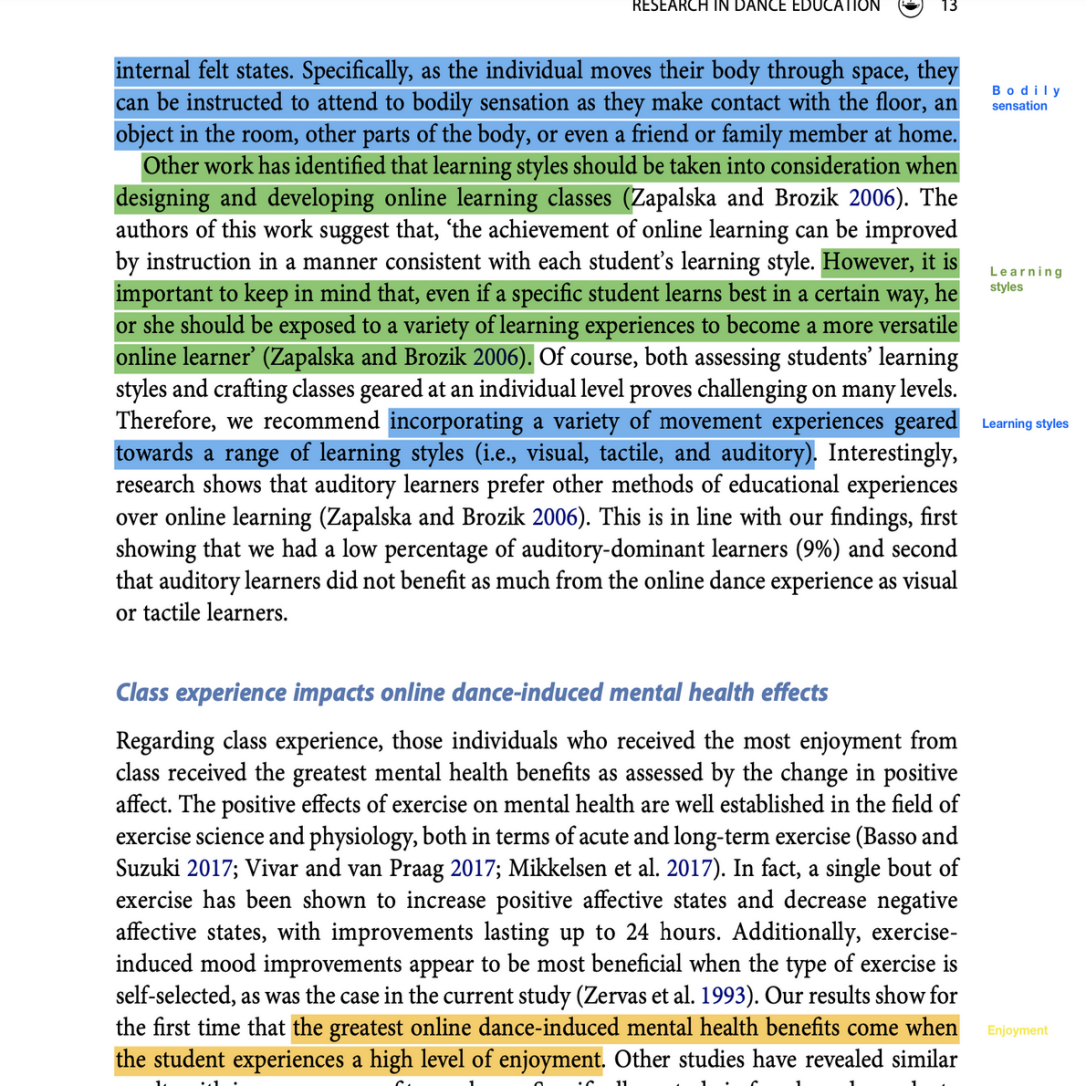
Example of line-by-line color coding [2].

Specifically, the first analysis stage involved line-by-line color coding of creatively adapted working methodology by 1 reviewer (GG). Coding was inductive, with the set of codes expanded as additional studies were added [30]. At least one code was assigned to all statements related to creatively adapted working methods. However, statements often had multiple codes. The preliminary codes were discussed and refined by the review team (GG, DJH-S, and REKC). Example codes included “self-touch exercises” and “interested patients.” An example of line-by-line coding can be found in Figure 1.

In the second stage, groups of related codes were identified and combined into broader descriptive themes by 1 reviewer (GG) and cross-checked by another reviewer (DJH-S). To ensure coherence, the process involved repeated reference to the papers from which the codes were derived. The third synthesis stage involved comparing the descriptive themes across fields and analyzing findings in relation to overarching topics.

### Role of the Core Research Team

We considered and acknowledged the influence of our individual perspectives on the findings and their presentation. We are all UK-based researchers, with GG and JW having a background in the creative arts, currently undertaking postgraduate training in the arts, and having qualitative research experience. REKC has a background in the creative arts and sociology and is currently an assistant research professor at Coventry University. DJH-S is a clinician-academic with a background in osteopathy and clinical pain research and an expert in systematic review methodology. All researchers individually led projects on web-based performance arts, web-based community dance projects, or remote consultations for manual therapists, respectively, during the early phases of the COVID-19 pandemic.

## Results

### Description of the Included Studies

**Figure 2:**
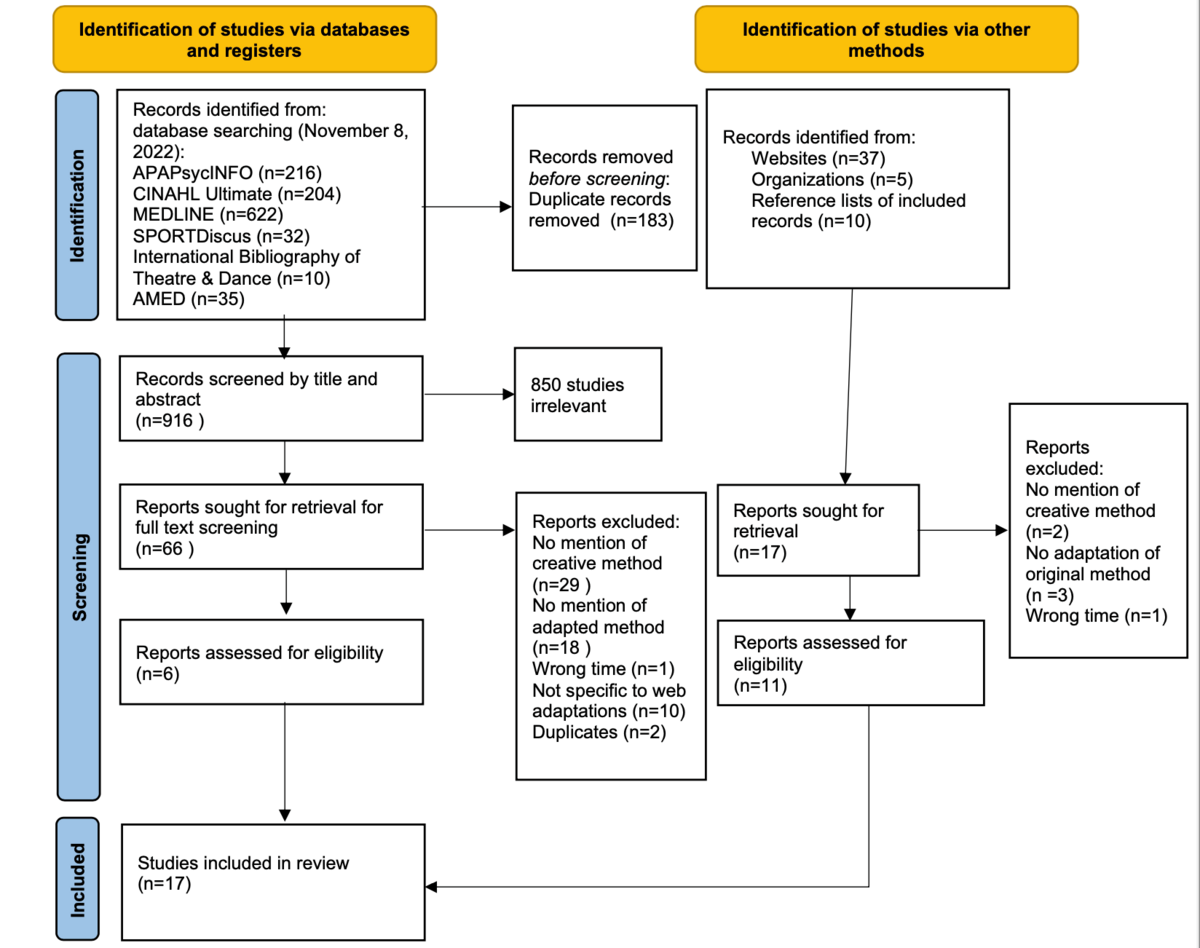
PRISMA (Preferred Reporting Items for Systematic Reviews and Meta-Analyses) flow diagram of the search strategy and study selection.

We identified 916 records from our database search after excluding duplicates. After screening the papers for titles and abstracts, of those 916 papers, 66 (7.2%) were accessed in full and screened again. A total of 6 papers met the review inclusion criteria. We identified a further 17 records by searching via the other aforementioned methods. After screening against the inclusion and exclusion criteria, of those 17 records, 11 (65%) met the inclusion criteria. Figure 2 [31] displays the PRISMA (Preferred Reporting Items for Systematic Reviews and Meta-Analyses) flow diagram of the search.

In total, 17 publications were included in this review. A detailed overview of the characteristics of the included studies can be found in Multimedia Appendix 2 [2,6,11,18,19,32-43]. All study designs were considered; however, only qualitative studies were found to be relevant after the screening process. All studies (17/17, 100%) were published between 2020 and 2022, with most studies (8/17, 47%) being published in 2022. Studies were mainly conducted in the United States (6/17, 35%), and most (5/17, 29%) were case studies. Furthermore, studies included a large range of fields, with art therapy (4/17, 24%) and dance (4/17, 24%) being the most included fields. The web-based platforms used for remote sessions varied. Table 1 provides a detailed description of these studies.

**Table 1:** Description of the studies (n=17).

			Studies, n (%)
**Year of publication**			
	2020	6 (35)	
	2021	3 (18)	
	2022	8 (47)	
**Country**			
	United States	6 (35)	
	United Kingdom	5 (29)	
	France	1 (6)	
	Israel	1 (6)	
	New Zealand	1 (6)	
	Australia	1 (6)	
	No specific location	2 (12)	
**Study design**			
	Case study	5 (29)	
	Interview study	3 (18)	
	Practice as research	2 (12)	
	Web-based survey	3 (18)	
	Personal observations	2 (12)	
	Reflective paper	1 (6)	
	Controlled nonrandomized study	1 (6)	
**Field of study**			
	Art therapy	4 (24)	
	Dance	4 (24)	
	Theater and drama	1 (6)	
	Body psychotherapy	1 (6)	
	Psychology	2 (12)	
	Integrative oncology	1 (6)	
	Nursing	1 (6)	
	Sport and fitness	1 (6)	
	Physiotherapy	1 (6)	
	Allied health	1 (6)	
**Web-based platform**			
	Zoom	5 (29)	
	WhatsApp	2 (12)	
	Combination of platforms	5 (29)	
	Nonspecified platform	5 (29)	

All methods were appropriate for qualitative designs, presenting clear aims, results, and implications. Lower-scoring papers usually provided less information on ethics, data collection, or research-participant relationship considerations. Generally, data collection methods were suitable, although sometimes important details were missing—authors rarely commented on their relationship with participants or the implementation of ethical procedures even though approval had been granted. Table 2 shows the quality assessment of the included studies using the CASP tool.

**Table 2:** Quality assessment of qualitative research using the Critical Appraisal Skills Programme tool for qualitative study appraisal.

Study	Clear statement of aims?	Qualitative methodology?	Appropriate research design?	Appropriate recruitment strategy?	Suitable data collection?	Research-participant relationship considered?	Ethical issues considered?	Rigorous data analysis?	Clear findings?	Valuable to us?
Athanasiadi [32]	Yes	Yes	Yes	Yes	Yes	Yes	Cannot tell, unclear	Yes	Yes	Yes
Ben-Arye et al [6]	Yes	Yes	Yes	Yes	Yes	Cannot tell, unclear	Yes	Yes	Yes	Yes
Datlen and Pandolfi [11]	Yes	Yes	Yes	Yes	Cannot tell, unclear	Cannot tell, unclear	Yes	No	Yes	Yes
Eve [39]	Yes	Yes	Yes	Yes	Yes	Cannot tell, unclear	Cannot tell, unclear	Cannot tell, unclear	Yes	Yes
Feniger-Schaal et al [36]	Yes	Yes	Yes	Yes	Yes	Yes	Cannot tell, unclear	Yes	Yes	Yes
Gauhe [35]	Yes	Yes	Yes	Yes	Yes	Cannot tell, unclear	Cannot tell, unclear	Yes	Yes	Yes
Grant et al [33]	Yes	Yes	Yes	Yes	Yes	Yes	Yes	Yes	Yes	Cannot tell, unclear
Karam and Naguib [41]	Yes	Yes	Yes	Yes	Yes	Yes	Yes	Yes	Yes	Yes
Lord [34]	Cannot tell, unclear	Yes	Yes	Yes	Yes	Yes	Yes	Yes	Yes	Yes
Malliaras et al [19]	Yes	Yes	Yes	Yes	Yes	Cannot tell, unclear	Cannot tell, unclear	Yes	Yes	Yes
Moloney et al [43]	Yes	Yes	Yes	Yes	Cannot tell, unclear	Yes	Yes	Cannot tell, unclear	Yes	Yes
Rugh et al [2]	Yes	Yes	Yes	Yes	Yes	Cannot tell, unclear	Cannot tell, unclear	Yes	Yes	Yes
Snyder [42]	Cannot tell, unclear	Yes	Cannot tell, unclear	Yes	Yes	Cannot tell, unclear	Cannot tell, unclear	Yes	Yes	Yes
Sieradzki and LeMarquand [37]	Cannot tell, unclear	Yes	Yes	Yes	Yes	Cannot tell, unclear	Cannot tell, unclear	Yes	Cannot tell, unclear	Yes
Thorpe et al [18]	Yes	Yes	Yes	Yes	Yes	Yes	Yes	Yes	Yes	Yes
Usiskin and Lloyd [38]	Yes	Yes	Yes	Yes	Cannot tell, unclear	Cannot tell, unclear	Yes	Yes	Yes	Yes
Weaver et al [40]	Yes	Yes	Yes	Yes	Yes	Yes	Cannot tell, unclear	Yes	Yes	Yes

In total, 53% (9/17) of the studies mentioned the number of participants, with a median of 17 (IQR 7.5-622) participants per study. The number of participants ranged from a minimum of 2 to a maximum of 15,334. Information about the study populations of all the included studies and their number of participants can be found in Table 3.

**Table 3:** Demographics—study population^a^.

Study	Study population	Participants^b^ (N=15,334), n (%)
Athanasiadi [32]	Audience members	NR^c^
Ben-Arye et al [6]	Patients undergoing chemotherapy	56 (0.37)
Datlen and Pandolfi [11]	Young adults with learning disabilities	5 (0.03)
Eve [39]	Web-based body psychotherapists	NR
Feniger-Schaal et al [36]	Art therapists offering telehealth	15,334 (100)
Gauhe [35]	Audience members	NR
Grant et al [33]	Physiotherapists who work with children	10 (0.07)
Karam and Naguib [41]	Audience members	NR
Lord [34]	A couple in couple therapy	2 (0.01)
Malliaras et al [19]	Allied health clinicians treating people with musculoskeletal conditions	1185 (7.73)
Moloney et al [43]	Men with learning disabilities	NR
Rugh et al [2]	Participants aged ≥18 y capable of engaging in physical activity	59 (0.38)
Snyder [42]	Children and teenagers with a variety of mental health issues	NR
Sieradzki and LeMarquand [37]	Dance students	NR
Thorpe et al [18]	Women working in the sport and fitness industry	17 (0.11)
Usiskin and Lloyd [38]	People displaced because of conflict, persecution, and poverty	NR
Weaver et al [40]	Adult-trained hospice nurses	15 (0.1)

^a^A total of 47% (8/17) of the studies had unspecified participant numbers.

^b^Median 17 (IQR 7.5-622) participants.

^c^NR: not reported.

### Qualitative Results

#### Overview

Creatively adapted working methods to suit the new web-based format were identified and grouped into 5 recurring themes that are described in the following sections and summarized in Multimedia Appendix 3 [2,6,11,18,19,32-43]. Studies contributing to each theme are shown in Multimedia Appendix 4 [2,6,11,18,19,32-43]. A minimum of 29% (5/17) of the studies contributed to each theme. The themes were cross-field adaptations, adapted working methods for sensorial experiences and nonphysical connections, creative methods that made use of materials or additional tools, creative use of web-based platform tools or additional technologies, and creative methods that required preparation. These themes will be discussed in the following sections. “Original methods that underwent adaptation” was another analysis theme but will only be presented as part of the summary table and can be found in Multimedia Appendix 3 to contextualize findings for readers.

Although there may be some overlap between the themes, each offers unique insights into the innovative responses adopted during the pandemic. They all explore various methods used by practitioners to recreate a sense of touch and connection on the web. The themes serve as a way to categorize these methods, although some could be combined or interlinked with one another. For instance, an arts therapy group used different objects in participants’ homes while simultaneously connecting through the WhatsApp web-based platform. They shared their creative outcomes as part of a web-based WhatsApp web-gallery. In this case, both materials and technology played a role in fostering connection and creating a sense of touch despite the physical distance, so this method informed 2 different themes. Table 4 provides an overview of the themes along with their descriptions and examples.

**Table 4:** Overview of themes.

Theme	Description	Example
Adapted working methods—cross-field methods	Implementation of methods from other fields to adapt practice to the new web-based format	An oncology program used breathing exercises as part of their web-based treatment plan.
Adapted working methods for sensorial experiences and nonphysical connections	Collection of methods addressing how sensorial experiences and nonbodily connections can be reproduced on the web	Practitioners used imagination and visualization to replicate the sense of touch.
Creative methods using materials or additional tools	Methods involving physical objects, materials, or digital tools to enhance web-based sessions and overcome the felt distance via the screens	Using a foam roller during a couple therapy session for a trust-based exercise
Creative use of web-based platform tools or additional technologies	Digital methods implemented to enhance the web-based experience for participants	Using the gallery feature of WhatsApp as an internet-based gallery
Creative methods requiring previous preparation of practitioners or participants	Methods requiring preparation by the practitioner or participant before web-based sessions	For a web-based performance, participants had to hide underneath a blanket before joining.

Throughout the following sections, we present participants’ and practitioners’ experiences along with the aforementioned themes of creatively adapted working methods where relevant.

#### Adapted Working Methods: Cross-Field Methods

A total of 29% (5/17) of the included studies presented creative working methods from other fields to adapt practice to the new web-based format. These techniques included breathing exercises, exercises inspired by meditation, somatic movements, body screening exercises, and other mind-body or spiritual approaches [6,19,32-34]. These techniques required participants to become active and focus on their internal states rather than on external feelings or perceptions:

Inspired by meditation activities to shift the body’s attention to its relationship with the surroundings and to re-shift the body’s attention to its sensorial imagination...[32]

By encouraging participants to self-manage their internal states and report back, allied health practitioners’ role shifted from their traditional role as “fixers” to that of coaches [19]. This shift required participants to become active and engaged, especially when trying techniques that focused on “felt” or “sensorial” experiences [19,34]:

To also convince patients that many musculoskeletal conditions can be self-managed by exercise and movement rather than manual and electro therapy [19].

Some of these methods, such as breathing exercises, also required the support of a caregiver or household member, triggering new forms of communication and ways to feel connected to one another, for example, during web-based couple therapy:

The physical contact and shared breath work that back-to-back communication offers can be grounding, supportive, and soothing for both members of the couple [34].

Notably, only 1 study from integrative oncology specified that experts from other fields were involved in delivering these techniques borrowed from other fields as part of their web-based sessions [6]. In all other studies (16/17, 94%), it was unclear whether experts from other fields were involved in either the planning or implementation of cross-field methods. Nevertheless, practitioners appreciated the support of in-person caregivers or other therapists performing home visits as they could be instructed to help with the treatment even though they were not themselves considered experts [33].

#### Adapted Working Methods to Recreate Sensorial Experiences and Nonphysical Connections

Many practitioners chose to include self-guided and self-administered exercises involving self-touch, self-massage, self-holding, self-acupressure, self-acupuncture, self-delivered palpation, and self-administered manual touch therapies to address the challenge of not being able to touch participants directly during web-based sessions [6,19,35]. Most studies (15/17, 88%) mentioned that participants enjoyed learning new skills and that they were open to self-treatment or willing to adapt. However, some participants found it difficult to adapt. For example, patients of manual therapists were worried about self-administering touch-involving exercises, art therapists’ patients often mentioned the lack of personal connections because of screens as a barrier [6,11,19], and dance or fitness class participants missed contact-based corrections of movements or postures [2,18]. Practitioners welcomed having to improve their communication skills to offer precise and direct instructions via the screen [19]. Nevertheless, practitioners also often mentioned the lack of support or training when having to guide touch-based exercises. Consequently, they highlighted the need to develop new working methods and adequate training [6,19,28,32,36,37].

Another way to deal with the challenge of “no touch” on the web was to ask carers, family members, or housemates to support the treatment by administering guided touch-involving methods. For example, they were asked to locate acupressure points or report back about how a body area felt. Furthermore, to enhance treatments, people present at the participant’s location were often encouraged to take part in tasks such as relaxation exercises or to sit back-to-back with the participant to show support and enable physical connections [6,33,34].

Some practitioners also chose to involve objects or materials to create sensorial opportunities for their participants [2,11,38]. Touching objects or props and even other household members were methods used to recall past encounters and evoke sensorial memories. Especially when other household members were not present, pushing or leaning against walls was used as a method to recreate touch-like sensations and physical boundaries [39]. A total of 12% (2/17) of the studies made use of different scents while encouraging their participants to touch objects such as a blanket or the surrounding environment to draw associations between the sensations of the body at a specific location in the room and the memory of a sensation that the scent triggered [32,39]. For example, an apple spice scent placed inside a prearranged safe environment brought about a sense of familiarity and comfort, which reinforced the experience of an enclosed space and a state of equilibrium [32].

Art therapists mentioned that some materials were particularly impactful when creating sensorial experiences for participants, such as clay or blankets, towels, and pillows [11,39]. The authors stated that these objects were used to “contain,” “center,” and “move” energy. By manipulating these objects with their eyes closed, sensory experiences were triggered for participants that related to past experiences [39]:

With a loss of person-to-person touch due to the distancing measures following COVID-19, the sensorial opportunities provided by the art materials, particularly clay, have become even more of a consideration when planning the session [11].

Art therapist

Finally, many practitioners from different fields, such as dance, art therapy, and theater, chose imagination as a tool to let participants re-engage with their sense of touch. To support participants in imagining touch-like sensations, practitioners used different methods. These included the use of guided imagery or voice recordings involving sensory imagery and sounds of objects touching [32,35,37].

Regarding the lack of a shared physical space, creating web-based spaces for people to connect and build communities was an effective way of dealing with loneliness or separation anxieties. In this case, screens and new technologies offered participants new ways to connect with friends, colleagues, or other participants, producing new sensory experiences of touch, connection, and care:

Because even though you are not physically there with each other, you can still push each other through the screen. And just knowing that there are people doing the same thing as you that are trying to work hard through this time was nice [18].

Football athlete taking part in study about online fitness classes

Another creative way to connect with participants on the web was to inquire about their emotions, feelings, hobbies, experiences, or senses (eg, “What is your heart telling you?” and “What are you sensing in your room?”) [40].

**Figure 3:**
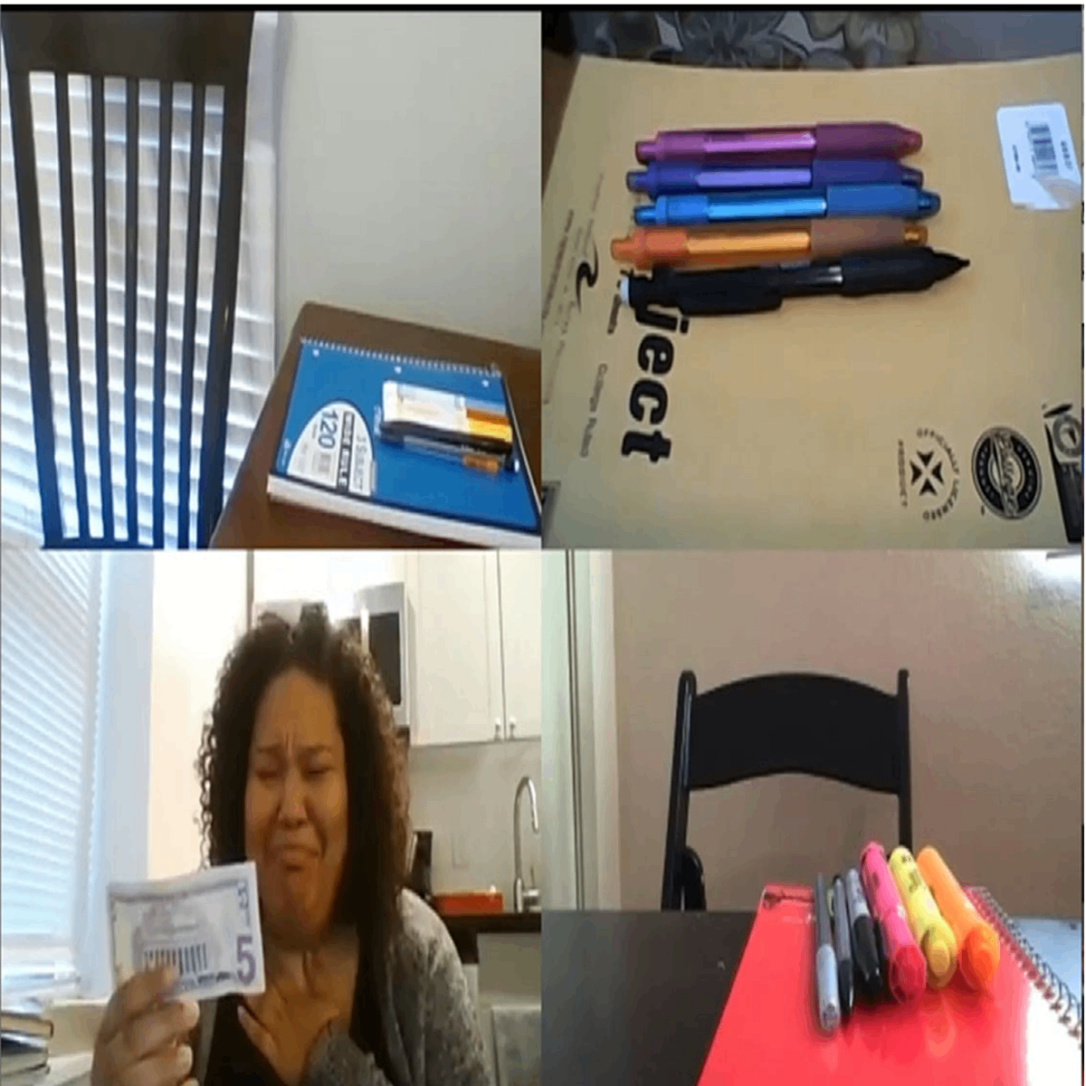
Illustration of a method to create the sensation of a shared space on the web. Image reproduced from Karam and Naguib [41].

Furthermore, by covering and uncovering the camera to explore transparency, opacity, closeness, or distance, theater practitioners tried to address social distance and physical separation [38]. During web-based theater performances, artists chose to divide the screen into 4 active panes, but only 1 was occupied by an actor. The other panes displayed objects or materials relevant to the actor’s scene. The other actors were not visible on the screens, but they were audible. This created a sense of coexistence even though all artists were in separate locations [41]. Figure 3 [41] shows a visual demonstration of the 4 panes.

#### Creative Methods That Made Use of Materials or Additional Tools

All practitioners made use of some materials, objects, or additional tools to creatively adapt to the new web-based format and engage their participants effectively.

Most practitioners made use of digitally available resources such as diagrams, photographs, or educational materials, whereas others asked participants to look for materials or objects available at their own locations [2,6,18,33,34,36-38,42]. The sharing of personal items helped practitioners connect personally with their participants. In addition, practitioners believed that the personal environment of their participants helped them foster imagination and helped make them more comfortable:

Sharing things from the environment can bridge the gap between the physical distance and the separate spaces [36].

In art therapy, practitioners used materials from patients’ homes to make costumes for digital role-plays. In dance training and fitness, they encouraged participants to share personal materials such as photographs [18]. Furthermore, practitioners encouraged making art from domestic objects or available natural materials [18,36-38].

Engaging many participants across the globe, a project initiated the creation of a digital quilt. Participants were invited to create a square with materials available in their own homes in response to social and political concerns:

It forms “a patchwork narrative that seeks to help cope with personal feelings of anxiety, while building empathy for others” experiences [38].

Art therapist

Practitioners appreciated being able to work with participants from different areas, making their sessions more accessible to a larger population without geographic or temporal barriers [11,32,34,35,38-41].

One way for participants to choose materials or objects in their homes was to create a treasure hunt as part of web-based fitness classes. In this case, participants were asked to, for example, find an object that helped them through the lockdown and share it with the group [18]. The sharing of personal items fostered connections with other participants and practitioners.

In a few cases, practitioners included objects from their own homes, with one study mentioning the use of a doll as a communication tool to model what the physiotherapists wanted the parents or caregivers to do with their children [33].

For some practitioners, it was relevant to send materials to participants’ homes to ensure equal accessibility or enable group work with similar materials [11]. For example, to take part in a web-based multisensory performance, participants were sent a box that included a score, a booklet, 2 soundtracks, 2 jars with perfumes in solid state, and a blindfold [32].

Apart from using objects or materials, practitioners included creative methods to keep their participants physically and mentally engaged. During a web-based couple therapy session, participants were asked to balance on a foam roller while being supported by their partner to foster trust [34]. Allied health clinicians often included walking and running exercises for patients to perform in their own time [19], and art therapists provided seated stretches or “freeze dances” to keep their participants actively engaged and emotionally aware via a screen [42]. In one case, physiotherapists started singing to their underage patients to keep their attention:

She (mum) was able to put the laptop with my face on it right in front of him. And that was really interesting that this child could actually enjoy that—I was singing to him from the computer [33].

Physiotherapist treating children online

To further enhance the experience of all participants during web-based sessions, one study also pointed to the importance of acknowledging different learning styles. Visual, auditory, and tactile learners all experience web-based working methods differently, with visual learners thought to benefit the most from web-based sessions as they are more focused on the visual aspect of web-based platforms and more engaged with the practitioner. However, one study found that tactile learners experienced their biggest gains in their affective state and auditory learners generally preferred in-person methods over web-based learning. Therefore, when preparing web-based sessions, practitioners might be well advised to consider a variety of exercises geared toward a range of learning styles (ie, visual, tactile, and auditory) [2].

#### Creative Use of Web-Based Platform Tools or Additional Technologies

Practitioners often used the tools available on different web-based platforms in creative ways to decrease the otherwise felt distance via screens and create personal connections on the web.

For example, during web-based dance sessions, practitioners used the spatial framing of the Zoom (Zoom Video Communications) screen as an esthetically engaging teaching tool. They played with proximity and distance as well as different angles or framing:

Drawing on dance film techniques such as perspective, proximity/distance, angles and framing allowed us to capitalize on the unique and novel role of the camera in online learning versus trying to make the experience exactly like an in-person class [2].

Similarly, the screen was turned into a theater where the performer could disappear and only share certain parts of the body or preselected objects in a certain order to create a storyline [36,38].

Furthermore, platforms were used to share videos, photos, documents, and links to additional web-based tools, including YouTube videos. A creative way of using a web-based platform was to use the gallery feature of WhatsApp as an internet-based gallery, echoing the original approach of exhibiting artworks [11]. Furthermore, internet-based backgrounds were used to enhance the esthetic experience of participants [36].

Going beyond the features available on the web-based platform Zoom, screen recordings were used as film material to create collage films [35], and TikTok’s video editing tools were used to create essays in the form of short videos:

They rose to the challenge, whether it was connecting the everyday movements of Yvonne Rainer to washing hands, or dueting with pets to demonstrate the ingenuity of Alwin Nikolais and his use of props [37].

Dance teacher talking about their students

To engage participants more directly, the chat function of web-based platforms such as Zoom or Teams (Microsoft Corporation) was used, where participants were encouraged to chat, use emojis, share emotions, or participate in polling [41]. Furthermore, TikTok’s “duet” or “react” functions were used to add on to other participants’ uploaded videos:

Beyond their singular contributions, the moments when they could “duet” or “react” to one another kept the collaborative aspect of our class alive even while we physically could not dance with one another [37].

Dance teacher talking about their students

Regarding group sessions, the breakout room feature on the web-based platform Zoom was a creative way to keep participants engaged and enable separate groups to work together. Furthermore, an internet-based foyer after web-based performances allowed participants to connect and share their experiences:

...not only sharing their experience but also becoming actively curious about other people’s experiences, critically evaluating the work, and more importantly voicing their concerns and/or suggestions [32].

Another successful way to engage participants on the web was to use additional software tools such as Adobe, Canva, PowerPoint, network games, and whiteboards to make art together or engage groups of people in one activity [36,42].

Sometimes, additional equipment was used to enhance the experience. Connecting external cameras allowed for a larger field of vision, and software tools such as QLab were used for a web-based theater production to control sound and video effects. A remote-control software was also used so that a member of the technical crew could log into another person’s computer to control it over a distance [41,43]. During hybrid art therapy sessions, a projector and screen were used at the front of a room with dual webcam coverage to help create context and maintain flow for participants in other locations [43].

Regarding the use of technology, many participants from different fields were worried about not being able to use new technologies without previous instruction, and some were not able to do so because of a lack of technology available in their homes. This highlighted inequalities and the need to secure further funding or adequate equipment for web-based sessions. In contrast, whenever participants had access to the right technology and previous knowledge, many accepted the use of new technology as it offered new solutions and they sometimes acquired new skills [2,6,11,19,33,38,42,43].

Practitioners especially mentioned many challenges regarding the use of technology, such as screen fatigue, technological issues, limitations of the camera, distraction, and the self-view being problematic. However, most practitioners agreed that technology enabled them to continue with their web-based sessions. They also felt that they were largely able to read facial expressions and body language via the screen [2,6,11,19,33,34,37,38,40].

#### Creative Methods Requiring Previous Preparation of Practitioners or Participants

Some creatively adapted working methods required the practitioner or participant to prepare before the actual web-based session. Preparing participants, family members, or caregivers for specific telehealth services and creative methods before the web-based session ensured participant safety, fewer interruptions, and clearer expectations [33,35]. For example, a web-based multisensory performance delivered written instructions and sketches to the participants to prepare the room (eg, clear the space to move around) and look for additional materials in advance of the actual performance [32]. Another web-based performance asked participants to prepare and set up their phone on their bed to then listen to sound recordings underneath a blanket [35].

Practitioners from health care reviewed preappointment medical imaging such as magnetic resonance images, and art therapists used survey-style questionnaires for assessments before the web-based session. This allowed practitioners to prepare for the sessions in advance. These ideas were specifically added for their web-based practice and were not necessarily included in in-person sessions [19,42].

Furthermore, practitioners sometimes had to prepare additional digital materials, for example, to guide self-treatment or offer video documentations about how to make specific materials such as dough to then use during their next web-based sessions:

I videoed myself explaining how to make salt dough to make play dough...I sent it and asked the client to prepare it in advance [36].

Art therapist

In some instances, such as during web-based theater performances, participants were also invited to change their setting on the platform they were using during the web-based session; for example, they had to keep their screens on or off, keep their microphones turned on or off, or choose a certain internet-based background. All these considerations helped practitioners ensure fewer disruptions during their sessions and create a better web-based experience for their participants [38,43].

## Discussion

### Principal Findings

Many touch-based field practitioners have developed or implemented creative working methods since the beginning of the COVID-19 pandemic to adapt their in-person sessions to the new web-based format, including physiotherapists, creative arts practitioners, art therapists, and fitness coaches. Our review showed that each field implemented unique and innovative ways of working, which enriched participants’ experiences during a difficult time.

Some professions implemented similar working methods on the web despite considerable differences in their offline ways of working. For example, art therapists, dance artists, integrative oncologists, and allied health clinicians all incorporated self-treatments. Furthermore, art therapists, fitness instructors, dance artists, psychologists, and physiotherapists all used materials or objects from participants’ homes to creatively involve people over a distance.

Other methods were more field specific, such as creating an internet-based “foyer” after a web-based multisensory performance. However, these methods may still be transferable to other fields in the future. A web-based session of a group pain management program might, for example, equally benefit from an internet-based foyer (or “waiting room”) before or after sessions aiming at recreating the informal waiting room experience remotely to facilitate the informal meeting of patients with similar symptoms, the exchange of personal experiences regarding the therapy program, and the finding of peer support. Furthermore, creating a digital patchwork of art in response to personal experiences, hopes, or fears might lend itself not only to art therapy sessions but also to patients of integrative oncology who often must deal with their feelings alone [44].

Using voice recordings that include sensory imagery can help remember touch and awaken sensorial experiences. This method could benefit not only dance artists and their audiences but also patients who feel disconnected from their senses and seek support from manual therapists or psychotherapists. Describing any of the 5 human senses is categorized as sensory imagery, a commonly used stylistic device in all forms of literature, where it helps create mood and enables the reader to picture the story’s setting [45]. Sensory imagery instructions such as “This might feel like cold water washing over you” or “My cold hand is resting on your warm shoulder, it will feel heavy” can allow participants to recall sensorial experiences by accessing their haptic memory, especially relevant during a time when actual physical contact is limited [46].

Going beyond the scope of this review, other innovative in-person methods that were developed long before the COVID-19 pandemic could equally lend themselves to the new web-based format. For example, animal-assisted therapies, the implementation of different odors to evoke memory, and the use of humor in health care have been ways to manage patients’ stress and anxiety levels [47-49]. Notably, the stroking of pets can reduce anxiety levels in patients even if they are not considered animal lovers [50]. Furthermore, a study found that any odor that for a given individual evokes a happy autobiographical memory can have a beneficial effect on their health [48]. Finally, the use of objects can enhance treatment during speech and language therapy sessions for children [51]. Although these methods do not inherently hinge on face-to-face interactions, their successful adaptation for web-based sessions and other eHealth approaches should be undertaken with due consideration of the specific therapy and context, mindful of practical constraints that may arise. These innovative ways of working could be especially impactful for practitioners who experience the lack of physical contact as a barrier or who struggle to connect with their participants on a personal level during web-based sessions. The potential for borrowing innovative working methods from diverse fields warrants further exploration. If found to be practicable, this approach may underscore the value of transcending professional boundaries, particularly within the digital realm.

The innovative working methods mentioned in this review incorporate a lot of “humanness” and emotion into one’s work, which has often been considered lacking when working on the web [52]. The physical risk of such methods is relatively low. However, the expectations of participants may have to be set accordingly. Particularly in health care, patients may have clear expectations that can conflict with more creative adaptations [53-55]. However, “a good match between patients’ expectations and outcomes has shown to improve patients’ satisfaction” [55]. The fact that art therapists were so creative may be due to their training, but nonexistent expectations or an open-mindedness of their participants might have also influenced their risk taking when implementing new creative methods [56]. Therefore, it seems important to set accurate expectations and explain possible new, creative working methods to patients before a web-based telehealth session.

Apart from setting accurate expectations, it is important to acknowledge the different contexts of health care settings as practitioners working in public health care systems often do not have enough time to implement creative, sometimes time-consuming methods. Therefore, the main application of the aforementioned methods will likely be for private practitioners, longer sessions in public health care systems. For instance, digital therapeutics can leverage prerecorded audio files accessible through an app to enhance treatment plans. These audio files could provide patients with guided ways to imagine and visualize touch. By incorporating this feature, digital therapeutics can offer a more immersive and effective experience for patients, contributing to improved therapeutic outcomes.

The significance of touch in hands-on fields such as manual therapy, art therapy, psychology, fitness coaching, dance, integrative oncology, and physiotherapy justifies a collective effort to develop practical methods that transcend physical barriers and promote accessibility, including web-based touch-based practices. Web-based interventions are especially relevant for individuals seeking touch-based benefits while also desiring greater agency over their experiences. The web-based format empowers participants to engage in touch-based interventions from the comfort and privacy of their own homes. Interestingly, some individuals may find that the physical distance in web-based sessions paradoxically fosters a stronger sense of connectedness. Web-based touch-based practices hold particular promise for individuals facing barriers to in-person interventions, such as geographical constraints, physical disabilities, or other circumstances that limit face-to-face sessions. Therefore, this review invites practitioners from diverse fields to draw inspiration from the innovative methods discussed and explore their applicability to their own web-based practices.

Furthermore, when finding it difficult to imagine how field-specific working methods could be adapted to suit someone’s own practice, we recommend reaching out to experts in the field to find creative ways of adapting or incorporating cross-field methods. Health practitioners, for example, could work closely with yoga practitioners when wanting to implement breathing exercises during their web-based sessions to ensure that their techniques are correctly applied and beneficial. Involving field-specific experts during the development phase of new working methods will arguably be key to ensuring successful implementation.

As seen in the past, collaborations across fields have often been seminal in developing innovative ways of working and ensuring a more tailored support for participants. Several studies have revealed the immense benefits of integrating the arts into health-related issues [57-59]. For example, arts engagement in childhood can support child development, enhancing mother-infant bonding, speech and language acquisition, and educational attainment [60,61]. Regarding web-based ways of working, we similarly believe that collaboration and exchange between fields is key in the future to learn from each other’s successes or challenges. Only by stepping out of their comfort zones will practitioners from different fields be able to develop original and seminal working methods for the digital realm.

### Strengths and Limitations

The strengths of this study include the fact that it is the first comprehensive qualitative synthesis to focus on creatively adapted working methods in touch-based fields to suit the new web-based format, offering some ideas about how to adapt in-person working methods across fields. As a multidisciplinary team, we conducted a comprehensive literature search and thematic synthesis to identify repeated themes across studies with regard to creative working methods and participants’ and practitioners’ experiences when working on the web. Our concurrent presentation of creatively adapted working methods as well as the challenges and advantages of participants’ and practitioners’ experiences permitted a comprehensive understanding.

This systematic review has several limitations that should be considered when interpreting the results. First, the results were reliant on how primary data were interpreted and reported, thus potentially carrying over the primary studies’ biases and limitations. All studies (17/17, 100%) only comprised qualitative literature and did not include quantitative studies as none were relevant to our review. In addition, the reporting of touch-based activities in many screened and otherwise eligible articles was often lacking, preventing us from including these studies in the review. Another potential limitation of this review is the lack of analysis of sociodemographic or protected characteristics, which may limit the generalizability of the findings for marginalized groups and their experiences with web-based methods of health care. Furthermore, data extraction and quality appraisal were performed by one author, whereas the second author cross-checked the data against the original articles. Compared with duplicate extraction, this approach may have introduced discrepancies in the process. However, this was considered acceptable given the qualitative nature of the information (as opposed to numerical extractions, which may be more prone to extraction errors). However, the main analysis used only qualitative data, for which text passages were extracted with, arguably, little potential for error. Finally, the review focused on the impact of the COVID-19 pandemic on the practice of touch-based activities on the web and did not include studies conducted before the pandemic. Future research could consider incorporating these studies to provide a more comprehensive understanding of this subject.

### Conclusions

In conclusion, our systematic review provides evidence of the crucial role of innovation and adaptability in touch-based fields, specifically in terms of increasing accessibility and inclusivity. The results highlight the potential of new methods to enhance the participant experience and emphasize the need for further research to fully understand the long-term effects and sustainability of these adaptations. These modifications could potentially improve traditional methods and automated care, such as digital therapeutics. These findings have far-reaching implications for practitioners and researchers in touch-based fields, as well as for individuals who draw on these practices for their well-being. As such, we hope that this review will serve as a catalyst for continued exploration and innovation in this area and contribute to the development of accessible and inclusive touch-based practices for all.

## Appendix

Multimedia Appendix 1 Search strategies for all databases.

Multimedia Appendix 2 Characteristics of the studies included in the systematic review.

Multimedia Appendix 3 Summary of themes developed in the qualitative synthesis with regard to creative working methods.

Multimedia Appendix 4 Contribution of studies to the synthesis of creatively adapted working methods.

## Abbreviations

CASP: Critical Appraisal Skills Programme
PICO: Population, Intervention, Comparison, and Outcome
PRISMA: Preferred Reporting Items for Systematic Reviews and Meta-Analyses

## Data Availability

The data sets generated and analyzed during this study are available from the corresponding author upon reasonable request.
