# Correction: Creatively Adapting Touch-Based Practices to the Web Format During the COVID-19 Pandemic: Systematic Review

**DOI:** 10.2196/55713

**Published:** 2024-01-05

**Authors:** Greta Gauhe, Rosemary E Kostic Cisneros, Jade Ward, David J Hohenschurz-Schmidt

**Affiliations:** 1 Centre for Dance Research Coventry University Coventry United Kingdom; 2 Pain Group Department of Surgery & Cancer Imperial College London London United Kingdom; 3 Research Department University College of Osteopathy London United Kingdom

In “Creatively Adapting Touch-Based Practices to the Web Format During the COVID-19 Pandemic: Systematic Review” (J Med Internet Res 2023;25:e46355) the authors noted two errors:

1. In the originally published article, in Tables 2 and 3 the in-text citation for reference [34] appeared as:

Lord Ferguson et al [34]

This has been corrected to:

Lord [34]

2. In the Reference list of the originally published article, reference [34] appeared as follows:

Lord Ferguson S, Smith C, Kietmann J. Hands-off? Lessons from high-touch professionals about going virtual. Bus Horiz. 2022 May;65(3):303-13. Doi:10.1016/j.bushor.2021.03.002.

This has been corrected to:

Lord SA. COVID Couple Therapy: Telehealth and Somatic Action Techniques. Aust N Z J Fam Ther. 2022;43(2):197-209. doi:10.1002/anzf.1487

The correction will appear in the online version of the paper on the JMIR Publications website on January 05, 2023, together with the publication of this correction notice. Because this was made after submission to PubMed, PubMed Central, and other full-text repositories, the corrected article has also been resubmitted to those repositories.
