# Supplementary material for: Creatively Adapting Touch-Based Practices to the Web Format During the COVID-19 Pandemic: Systematic Review
Source: J Med Internet Res. 2023 Oct 26;25:e46355. doi: 10.2196/46355 (PMC10636621; doi:10.2196/46355)
Supplement: Multimedia Appendix 1 [file jmir_v25i1e46355_app1.docx]

**Search Strategies for Databases Search**

**Search strategy for Medline Database**

|  | **Search** **Terms** |
| --- | --- |
| **Population** | (MH "Midwifery") OR (MH "Nurse Midwives") OR (MH "Advanced Practice Nursing") OR (MH "Nurses, Community Health") OR (MH "Nurses, Male") OR (MH "Nurses, Neonatal") OR (MH "Nurses, Pediatric") OR (MH "Nurses, International") OR (MH "Nurses") OR (MH "Nurse's Role") OR (MH "Nursing Stations") OR (MH "Nursing Assistants") OR (MH "Nurses Improving Care for Health System Elders") OR (MH "Nurses, Public Health") OR (MH "International Council of Nurses") OR (MH "Practice Patterns, Nurses'") OR (MH "Licensed Practical Nurses") OR (MH "American Nurses' Association") OR birth attendant* OR (traditional birth attendant) OR labour support OR carer* OR (MH “family nurse practitioners”) OR pediatric* OR doula*  (MH "Therapeutics") OR (MH "Manual Communication") OR (MH "Sex Manuals") OR (MH "Musculoskeletal Manipulations") OR (MH "Neoadjuvant Therapy") OR Manual therapist* OR manual therapy OR manipulation therapy* OR reflexology OR rolfing OR craniosacral massage OR bodywork* OR acupressure OR motion therapy OR (motion therapist*) OR (MH "Osteopathic Physicians") OR (MH "Manipulation, Osteopathic") OR (MH "Osteopathic Medicine") OR (MH "Hospitals, Osteopathic") OR osteopathic OR osteopath* OR orthopedic OR (physical therapy) OR (soft tissue) OR kinesiology OR manipulative therapy OR manipulative therapies OR musculoskeletal OR musculoskeletal manipulations OR  (MH "Physical Therapy Modalities") OR (MH "Physical Therapists") OR (MH "Physical Therapy Specialty") OR (MH "Physical Therapist Assistants") OR Physiotherapy* OR physiotherapist* OR dry needling OR physical therapists OR physical therapist* OR orthopedic manipulation OR cryotherapy OR (occupational therapy) OR (mind-body therapies) OR chiropractic* OR (respiratory therapy) OR rehabilitation* OR (pain management) OR (exercise movement technique) OR (motion therapy) OR (muscle stretching exercise*) OR (resistance training) OR plyometric OR (aquatic therapy) OR (kinesiology applied) OR myofunctional therapy OR drainage OR endurance training OR (mirror movement*) OR  (MH "Psychology") OR (MH "Transference, Psychology") OR (MH "Reinforcement, Psychology") OR (MH "Psychology, Child") OR (MH "Transfer, Psychology") OR (MH "Psychology, Sports") OR Psychology* OR psychologist* OR cognitive psychology OR (cognitive science) OR (cognitive neuroscience*) OR (cognitive psychology) OR (psychology child) OR (behavioral psychology) OR psychiatry OR psychiatric* OR  Fitness coach* OR fitness teacher OR fitness* OR (physical educator*) OR (drill instructor*) OR fitness educator OR (personal fitness trainer*) OR personal coach OR (fitness manager) OR (physical functional performance) OR (physical endurance) OR  (Healthcare worker*) OR (personnel health) OR (healthcare provider*) OR (allied health personnel) OR nursing assistant* OR (pharmacy technician*) OR (physician assistant*) OR anatomist* OR anesthetist* OR (dental staff) OR dentist* OR hygienist* OR oral surgeon* OR orthodontist* OR (medical staff) OR pharmacists OR (physician executive*) OR physician* OR neurologist* OR cardiologist* OR dermatologist* OR oncologists* OR pediatrician* OR pathologist* OR radiologist* OR rheumatologist* OR urologist* OR surgeon* OR  Artist* OR (dance artist) OR dancer* OR actor*OR actress OR visual artist*OR ballerina*OR movement practitioner* OR art* OR choreographer* OR producer* OR (sound composer)OR (sound artist) OR composer* OR director* OR artistic director OR (rehearsal director) OR painter* OR  (MH "Physical Therapists") OR (MH "Art") OR (MH "Occupational Therapists") OR (MH "Language Arts") OR (MH "Art Therapy") OR (MH "Martial Arts") OR (MH "Physical Therapist Assistants") OR (MH "Science in the Arts") OR (MH "Sensory Art Therapies") OR (MH "Medicine in the Arts") OR (Art therapist) OR arts-therapies OR dance therapy OR psychotherapy* OR physical therapy* OR physical therapists OR therapeutics OR (sensory art therapy*)OR complementary therapies* OR (complementary therapy) OR (therapy dance) OR (color therapy*) OR play therapy OR music therapy OR expressive therapist |
| **AND**  **Intervention** | (Online OR remote OR digital* OR telehealth OR telemedicine OR (MH "Telemedicine") OR (mobile health) OR ehealth OR mhealth OR telecommunication* OR (MH "Remote Consultation") OR telepathology OR telerehabilitation OR teleradiology OR (remote consultation*) OR (online health) OR (video conference*) OR (online platform*) OR (virtual assessment*) OR virtual OR tele-creative art* OR teletherapy*) |
| **AND** | Touch* (MH "Touch") OR (MH "Touch Perception") OR (MH "Therapeutic Touch") |

**Search Strategy for APAPsych Database**

|  | **Search Terms** |
| --- | --- |
| Population | Midwives OR midwife OR midwifery* OR nurse* OR birth attendant* OR (traditional birth attendant) OR labour support OR carer*OR  family nurse practitioners OR pediatric* OR doula*  Manual therapist* OR manual therapy OR manipulation therapy* OR reflexology OR rolfing OR craniosacral massage OR bodywork* OR acupressure OR motion therapy OR (motion therapist*) OR osteopathic OR osteopath OR osteopaths OR orthopedic OR (physical therapy) OR (soft tissue) OR kinesiology OR manipulative therapy OR manipulative therapies OR musculoskeletal OR musculoskeletal manipulations OR  Physiotherapy* OR physiotherapist*OR dry needling OR physical therapists OR physical therapist*OR orthopedic manipulation OR cryotherapy OR (occupational therapy) OR (mind-body therapies)OR chiropractic*OR (respiratory therapy) OR rehabilitation* OR (pain management) OR (exercise movement technique) OR (motion therapy) OR (muscle stretching exercise*) OR (resistance training) OR plyometric OR (aquatic therapy) OR (kinesiology applied) OR myofunctional therapy OR drainage OR endurance training OR (mirror movement*) OR  Psychology* OR psychologist* OR cognitive psychology OR (cognitive science)OR (cognitive neuroscience*) OR (cognitive psychology) OR (psychology child) OR (behavioral psychology) OR psychiatry OR psychiatric* OR  Fitness coach* OR fitness teacher OR fitness* OR (physical educator*) OR (drill instructor*) OR fitness educator OR (personal fitness trainer*) OR personal coach OR (fitness manager) OR (physical functional performance) OR (physical endurance) OR  (Healthcare worker*) OR (personnel health) OR (healthcare provider*) OR (allied health personnel)OR nursing assistant*OR (pharmacy technician*) OR (physician assistant*) OR anatomist* OR anesthetist* OR (dental staff)OR dentist* OR hygienist* OR oral surgeon* OR orthodontist* OR (medical staff)OR pharmacists OR (physician executive*) OR physician* OR neurologist* OR cardiologist* OR dermatologist* OR oncologists* OR pediatrician* OR pathologist* OR radiologist* OR rheumatologist* OR urologist* OR surgeon* OR  Artist* OR (dance artist) OR dancer* OR actor* OR actress OR visual artist* OR ballerina* OR movement practitioner*OR art*OR choreographer*OR producer* OR (sound composer) OR (sound artist) OR composer* OR director* OR artistic director OR (rehearsal director) OR painter*OR  (Art therapist) OR arts-therapies OR dance therapy OR psychotherapy*OR physical therapy*OR physical therapists OR therapeutics OR (sensory art therapy*) OR complementary therapies* OR (complementary therapy) OR (therapy dance) OR (color therapy*) OR play therapy OR music therapy OR expressive therapist |
| **AND**  **Intervention** | (Online OR remote OR digital* OR telehealth OR telemedicine OR (mobile health) OR ehealth OR mhealth OR telecommunication* OR telepathology OR telerehabilitation OR teleradiology OR (remote consultation*) OR (online health) OR (video conference*) OR (online platform*) OR (virtual assessment*) OR virtual OR tele-creative art* OR teletherapy*) |
| **AND** | Touch* |

**Search Strategy for CINAHL (the Cumulative Index to Nursing and Allied Health Literature) Database**

|  | **Search Terms** |
| --- | --- |
| Population | Midwives OR midwife OR midwifery* OR nurse* OR birth attendant*OR (traditional birth attendant) OR labour support OR carer*OR (MH "Midwife Attitudes") OR (MH "Nurse Midwives") OR (MH "Midwives") OR (MH “doula”)  family nurse practitioners OR pediatric* OR doula*  Manual therapist* OR manual therapy OR manipulation therapy* (MH "Massage Therapists") OR (MH "Respiratory Therapists") OR (MH "Occupational Therapist Attitudes") OR (MH "Physical Therapist Attitudes") OR (MH "Recreational Therapists") OR (MH "Physical Therapists") OR (MH "Occupational Therapists") OR (MH "Craniosacral Therapists") OR reflexology OR rolfing OR craniosacral massage OR bodywork* OR acupressure OR motion therapy OR (motion therapist*) OR (MH "Osteopaths") OR (MH "Osteopathic Medicine") OR (MH "Manipulation, Osteopathic") OR (MH "Osteopathy") OR (MH "Manipulation, Orthopedic") OR "osteopath" OR osteopathic OR osteopath OR osteopaths OR orthopedic OR (physical therapy) OR (soft tissue) OR kinesiology OR manipulative therapy OR manipulative therapies OR musculoskeletal OR musculoskeletal manipulations OR  Physiotherapy* OR physiotherapist*OR dry needling OR physical therapists OR (MH "Physiotherapy Evidence Database") OR (MH "Physical Therapy Practice, Research-Based") OR (MH "Pediatric Physical Therapy") OR (MH "Physical Therapy") OR physical therapist* OR orthopedic manipulation OR cryotherapy OR (occupational therapy) OR (mind-body therapies)OR chiropractic* OR (respiratory therapy) OR rehabilitation* OR (pain management) OR (exercise movement technique) OR (motion therapy) OR (muscle stretching exercise*) OR (resistance training) OR plyometric OR (aquatic therapy) OR (kinesiology applied) OR myofunctional therapy OR drainage OR endurance training OR (mirror movement*) OR  Psychology* OR psychologist*OR cognitive psychology OR (cognitive science)OR (cognitive neuroscience*) OR (cognitive psychology) OR (psychology child) OR (MH "Psychology") OR (MH "Child Psychology") OR (MH "Psychology, Sports") OR (MH "Psychology, Occupational") OR (MH "Adaptation, Psychological") OR (MH "Human Needs (Psychology)") OR (MH "Religion and Psychology") OR (MH "Psychology, Social") OR (MH "Psychology, Applied") OR (MH "Psychology, Clinical") OR (MH "Positive Psychology") OR (MH "Health Psychology") OR (MH "Psychiatry") OR (MH "Child Psychiatry") OR (MH "Telepsychiatry") OR (MH "Social Workers") OR (MH "Psychiatric Service") OR (MH "Psychiatric Patients") OR (behavioral psychology) OR psychiatry OR psychiatric* OR  Fitness coach* OR fitness teacher OR fitness* OR (physical educator*)OR (drill instructor*) OR fitness educator OR (personal fitness trainer*) OR personal coach OR (fitness manager) OR (MH "Physical Fitness") OR (MH "Coaches, Athletic") OR (physical functional performance) OR (physical endurance) OR  (Healthcare worker*) OR (personnel health) OR (healthcare provider*) OR (allied health personnel)OR nursing assistant* OR (pharmacy technician*) OR (physician assistant*) OR anatomist* OR anesthetist* OR (dental staff)OR dentist* OR hygienist* OR oral surgeon* OR orthodontist* OR (medical staff)OR pharmacists OR (physician executive*) OR physician*OR neurologist* OR cardiologist*OR dermatologist* OR (MH "Dermatologists") OR oncologists* OR (MH “Oncologists”) OR pediatrician* OR (MH “pediatrician”) OR pathologist* OR (MH “pathologist”) OR radiologist* OR (MH “radiologist”) OR (MH “rheumatologist”) rheumatologist* OR urologist* OR (urologist*) OR surgeon* OR (MH “surgeon”) OR  Artist* OR (dance artist) OR dancer* OR ac tor*OR actress OR visual artist* OR ballerina*OR movement practitioner* OR art* OR choreographer* OR producer*OR (sound composer) OR (sound artist) OR composer* OR director* OR artistic director OR (rehearsal director) OR painter*OR  (Art therapist) OR (MH "Occupational Therapist Attitudes") OR (MH "Physical Therapist Attitudes") OR (MH "Respiratory Therapist Attitudes") OR (MH "Performing Arts") OR (MH "Respiratory Therapists") OR (MH "Massage Therapists") OR (MH "Physical Therapists") OR (MH "Occupational Therapists") OR (MH "Craniosacral Therapists") OR arts-therapies OR dance therapy OR psychotherapy*OR physical therapy*OR physical therapists OR therapeutics OR (sensory art therapy*) OR complementary therapies* OR (complementary therapy) OR (therapy dance) OR (color therapy*) OR play therapy OR music therapy OR expressive therapist |
| **AND**  **Intervention** | Online OR remote OR digital* OR telehealth OR telemedicine OR (mobile health) OR (MH "Remote Consultation") OR (MH "Videoconferencing") OR (MH "Telepathology") OR (MH "Teleconferencing") OR (MH "Rural Health Services") OR (MH "Rural Health Nursing") OR ehealth OR mhealth OR telecommunication* OR telepathology OR telerehabilitation OR teleradiology OR (remote consultation*) OR (online health) OR (video conference*)OR (online platform*) OR (virtual assessment*) OR virtual OR tele-creative art* OR teletherapy*) |
| **AND** | Touch* OR (MH "Touch") OR (MH "Therapeutic Touch") |

**Search Strategy for SportDiscus Database**

|  | **Search Terms** |
| --- | --- |
| Population | Midwives OR midwife OR midwifery* OR nurse* OR birth attendant* OR (traditional birth attendant) OR labour support OR carer* OR  family nurse practitioners OR pediatric* OR doula*  Manual therapist* OR manual therapy OR manipulation therapy* OR reflexology OR rolfing OR craniosacral massage OR bodywork* OR acupressure OR motion therapy OR (motion therapist*) OR osteopathic OR osteopath OR osteopaths OR orthopedic OR (physical therapy) OR (soft tissue) OR kinesiology OR manipulative therapy OR manipulative therapies OR musculoskeletal OR musculoskeletal manipulations OR  Physiotherapy* OR physiotherapist*OR dry needling OR physical therapists OR physical therapist*OR orthopedic manipulation OR cryotherapy OR (occupational therapy) OR (mind-body therapies)OR chiropractic*OR (respiratory therapy) OR rehabilitation* OR (pain management) OR (exercise movement technique) OR (motion therapy) OR (muscle stretching exercise*) OR (resistance training) OR plyometric OR (aquatic therapy) OR (kinesiology applied) OR myofunctional therapy OR drainage OR endurance training OR (mirror movement*) OR  Psychology* OR psychologist* OR cognitive psychology OR (cognitive science)OR (cognitive neuroscience*) OR (cognitive psychology) OR (psychology child) OR (behavioral psychology) OR psychiatry OR psychiatric* OR  Fitness coach* OR fitness teacher OR fitness* OR (physical educator*) OR (drill instructor*) OR fitness educator OR (personal fitness trainer*) OR personal coach OR (fitness manager) OR (physical functional performance) OR (physical endurance) OR  (Healthcare worker*) OR (personnel health) OR (healthcare provider*) OR (allied health personnel)OR nursing assistant*OR (pharmacy technician*) OR (physician assistant*) OR anatomist* OR anesthetist* OR (dental staff)OR dentist* OR hygienist* OR oral surgeon* OR orthodontist* OR (medical staff)OR pharmacists OR (physician executive*) OR physician* OR neurologist* OR cardiologist* OR dermatologist* OR oncologists* OR pediatrician* OR pathologist* OR radiologist* OR rheumatologist* OR urologist* OR surgeon* OR  Artist* OR (dance artist) OR dancer* OR actor* OR actress OR visual artist*OR ballerina* OR movement practitioner*OR art*OR choreographer*OR producer* OR (sound composer) OR (sound artist) OR composer* OR director* OR artistic director OR (rehearsal director) OR painter*OR  (Art therapist) OR arts-therapies OR dance therapy OR psychotherapy*OR physical therapy*OR physical therapists OR therapeutics OR (sensory art therapy*) OR complementary therapies* OR (complementary therapy) OR (therapy dance) OR (color therapy*) OR play therapy OR music therapy OR expressive therapist |
| **AND**  **Intervention** | (Online OR remote OR digital* OR telehealth OR telemedicine OR (mobile health) OR ehealth OR mhealth OR telecommunication* OR telepathology OR telerehabilitation OR teleradiology OR (remote consultation*) OR (online health) OR (video conference*) OR (online platform*) OR (virtual assessment*) OR virtual OR tele-creative art* OR teletherapy*) |
| **AND** | Touch* |

**Search Strategy for International Bibliography of Theatre & Dance Database**

|  | **Search Terms** |
| --- | --- |
| Population | Midwives OR midwife OR midwifery* OR nurse* OR birth attendant* OR (traditional birth attendant) OR labour support OR carer* OR  family nurse practitioners OR pediatric* OR doula*  Manual therapist* OR manual therapy OR manipulation therapy* OR reflexology OR rolfing OR craniosacral massage OR bodywork* OR acupressure OR motion therapy OR (motion therapist*) OR osteopathic OR osteopath OR osteopaths OR orthopedic OR (physical therapy) OR (soft tissue) OR kinesiology OR manipulative therapy OR manipulative therapies OR musculoskeletal OR musculoskeletal manipulations OR  Physiotherapy* OR physiotherapist*OR dry needling OR physical therapists OR physical therapist*OR orthopedic manipulation OR cryotherapy OR (occupational therapy) OR (mind-body therapies)OR chiropractic*OR (respiratory therapy) OR rehabilitation* OR (pain management) OR (exercise movement technique) OR (motion therapy) OR (muscle stretching exercise*) OR (resistance training) OR plyometric OR (aquatic therapy) OR (kinesiology applied) OR myofunctional therapy OR drainage OR endurance training OR (mirror movement*) OR  Psychology* OR psychologist* OR cognitive psychology OR (cognitive science)OR (cognitive neuroscience*) OR (cognitive psychology) OR (psychology child) OR (behavioral psychology) OR psychiatry OR psychiatric* OR  Fitness coach* OR fitness teacher OR fitness* OR (physical educator*) OR (drill instructor*) OR fitness educator OR (personal fitness trainer*) OR personal coach OR (fitness manager) OR (physical functional performance) OR (physical endurance) OR  (Healthcare worker*) OR (personnel health) OR (healthcare provider*) OR (allied health personnel)OR nursing assistant*OR (pharmacy technician*) OR (physician assistant*) OR anatomist* OR anesthetist* OR (dental staff)OR dentist* OR hygienist* OR oral surgeon* OR orthodontist* OR (medical staff)OR pharmacists OR (physician executive*) OR physician* OR neurologist* OR cardiologist* OR dermatologist* OR oncologists* OR pediatrician* OR pathologist* OR radiologist* OR rheumatologist* OR urologist* OR surgeon* OR  Artist* OR (dance artist) OR dancer* OR actor* OR actress OR visual artist*OR ballerina* OR movement practitioner*OR art*OR choreographer*OR producer* OR (sound composer) OR (sound artist) OR composer* OR director* OR artistic director OR (rehearsal director) OR painter*OR  (Art therapist) OR arts-therapies OR dance therapy OR psychotherapy*OR physical therapy*OR physical therapists OR therapeutics OR (sensory art therapy*) OR complementary therapies* OR (complementary therapy) OR (therapy dance) OR (color therapy*) OR play therapy OR music therapy OR expressive therapist |
| **AND**  **Intervention** | (Online OR remote OR digital* OR telehealth OR telemedicine OR (mobile health) OR ehealth OR mhealth OR telecommunication* OR telepathology OR telerehabilitation OR teleradiology OR (remote consultation*) OR (online health) OR (video conference*) OR (online platform*) OR (virtual assessment*) OR virtual OR tele-creative art* OR teletherapy*) |
| **AND** | Touch* |

**Search Strategy for AMED (the Allied and Complementary Medicine Database) Database**

|  | **Search Terms** |
| --- | --- |
| Population | Midwives OR midwife OR midwifery* OR nurse* OR birth attendant* OR (traditional birth attendant) OR labour support OR carer* OR  family nurse practitioners OR pediatric* OR doula*  Manual therapist* OR manual therapy OR manipulation therapy* OR reflexology OR rolfing OR craniosacral massage OR bodywork* OR acupressure OR motion therapy OR (motion therapist*) OR osteopathic OR osteopath OR osteopaths OR orthopedic OR (physical therapy) OR (soft tissue) OR kinesiology OR manipulative therapy OR manipulative therapies OR musculoskeletal OR musculoskeletal manipulations OR  Physiotherapy* OR physiotherapist*OR dry needling OR physical therapists OR physical therapist*OR orthopedic manipulation OR cryotherapy OR (occupational therapy) OR (mind-body therapies)OR chiropractic*OR (respiratory therapy) OR rehabilitation* OR (pain management) OR (exercise movement technique) OR (motion therapy) OR (muscle stretching exercise*) OR (resistance training) OR plyometric OR (aquatic therapy) OR (kinesiology applied) OR myofunctional therapy OR drainage OR endurance training OR (mirror movement*) OR  Psychology* OR psychologist* OR cognitive psychology OR (cognitive science)OR (cognitive neuroscience*) OR (cognitive psychology) OR (psychology child) OR (behavioral psychology) OR psychiatry OR psychiatric* OR  Fitness coach* OR fitness teacher OR fitness* OR (physical educator*) OR (drill instructor*) OR fitness educator OR (personal fitness trainer*) OR personal coach OR (fitness manager) OR (physical functional performance) OR (physical endurance) OR  (Healthcare worker*) OR (personnel health) OR (healthcare provider*) OR (allied health personnel)OR nursing assistant*OR (pharmacy technician*) OR (physician assistant*) OR anatomist* OR anesthetist* OR (dental staff)OR dentist* OR hygienist* OR oral surgeon* OR orthodontist* OR (medical staff)OR pharmacists OR (physician executive*) OR physician* OR neurologist* OR cardiologist* OR dermatologist* OR oncologists* OR pediatrician* OR pathologist* OR radiologist* OR rheumatologist* OR urologist* OR surgeon* OR  Artist* OR (dance artist) OR dancer* OR actor* OR actress OR visual artist*OR ballerina* OR movement practitioner*OR art*OR choreographer*OR producer* OR (sound composer) OR (sound artist) OR composer* OR director* OR artistic director OR (rehearsal director) OR painter*OR  (Art therapist) OR arts-therapies OR dance therapy OR psychotherapy* OR physical therapy* OR physical therapists OR therapeutics OR (sensory art therapy*) OR complementary therapies* OR (complementary therapy) OR (therapy dance) OR (color therapy*) OR play therapy OR music therapy OR expressive therapist |
| **AND**  **Intervention** | (Online OR remote OR digital* OR telehealth OR telemedicine OR (mobile health) OR ehealth OR mhealth OR telecommunication* OR telepathology OR telerehabilitation OR teleradiology OR (remote consultation*) OR (online health) OR (video conference*) OR (online platform*) OR (virtual assessment*) OR virtual OR tele-creative art* OR teletherapy*) |
| **AND** | Touch* |
